# Significance of melanin distribution in the epidermis for the protective effect against UV light

**DOI:** 10.1038/s41598-024-53941-0

**Published:** 2024-02-12

**Authors:** Daniela F. Zamudio Díaz, Loris Busch, Marius Kröger, Anna Lena Klein, Silke B. Lohan, Karsten R. Mewes, Lars Vierkotten, Christian Witzel, Sascha Rohn, Martina C. Meinke

**Affiliations:** 1 Department of Dermatology, Venereology and Allergology, Center of Experimental and Applied Cutaneous Physiology, Charité – Universitätsmedizin Berlin, Corporate Member of Freie Universität Berlin and Humboldt-Universität zu Berlin, Charitéplatz 1, 10117 Berlin, Germany; 2 Institute of Food Technology and Food Chemistry, Technische Universität Berlin, Gustav-Meyer-Allee 25, 13355 Berlin, Germany; 3 Department of Pharmaceutics and Biopharmaceutics, Philipps-Universität Marburg, Robert-Koch-Str. 4, 35032 Marburg, Germany; 4 Henkel AG & Co. KGaA, Henkelstr. 67, 40589 Düsseldorf, Germany

**Keywords:** Skin stem cells, Lasers, LEDs and light sources, Risk factors, DNA damage and repair

## Abstract

Melanin, the most abundant skin chromophore, is produced by melanocytes and is one of the key components responsible for mediating the skin’s response to ultraviolet radiation (UVR). Because of its antioxidant, radical scavenging, and broadband UV absorbing properties, melanin reduces the penetration of UVR into the nuclei of keratinocytes. Despite its long-established photoprotective role, there is evidence that melanin may also induce oxidative DNA damage in keratinocytes after UV exposure and therefore be involved in the development of melanoma. The present work aimed at evaluating the dependence of UV-induced DNA damage on melanin content and distribution, using reconstructed human epidermis (RHE) models. Tanned and light RHE were irradiated with a 233 nm UV-C LED source at 60 mJ/cm^2^ and a UV lamp at 3 mJ/cm^2^. Higher UV-mediated free radicals and DNA damage were detected in tanned RHE with significantly higher melanin content than in light RHE. The melanin distribution in the individual models can explain the lack of photoprotection. Fluorescence lifetime-based analysis and Fontana–Masson staining revealed a non-homogeneous distribution and absence of perinuclear melanin in the tanned RHE compared to the in vivo situation in humans. Extracellularly dispersed epidermal melanin interferes with photoprotection of the keratinocytes.

## Introduction

Although UVR is involved in the natural synthesis of vitamin D and endorphins, overexposure is considered a major risk factor for the development and progression of skin cancer due to several mutations caused by DNA damage in stem cells^1,2^. DNA damage in epidermal cells is mainly induced by direct absorption of UV-B radiation (280–320 nm) by pyrimidine bases, but also indirectly by radical formation after UV-A irradiation (320–400 nm)^1,3^. This results in the formation of the most ubiquitous DNA lesions—cyclobutane-pyrimidine dimers (CPD) and pyrimidine (6-4)-pyrimidone photoproducts (6-4PP)—along with single chain breaks and 8-oxo-7,8-dihydroguanine (8-oxoGua), respectively^4,5^.

Skin pigmentation has been considered the main photoprotective factor against UVR^6,7^, and ionizing radiation^8,9^ with many epidemiological studies showing a lower skin cancer incidence (20- to 60-fold) in dark skin (skin type VI) compared to light skin (skin types: I–II)^2^. The risk has often been attributed to differences in the content and composition of melanin in the skin^10^. Melanin, a complex biopolymer with condensed oxidized tyrosine as basis, is the most abundant skin chromophore, being presented as dark eumelanin and reddish pheomelanin^11,12^. Melanocytes synthesize and package melanin in the form of melanosomes and transfer it to adjacent keratinocytes, where the pigments arrange perinuclearly to protect the skin against UV-induced cell damage^11,13^. The distribution of melanin in a capsular structure over the keratinocyte nuclei is required to constitute a physical barrier that scatters, absorbs, and thus, reduces the penetration of UVR through the epidermis^14,15^. It is well known that photoprotective properties of melanin derive from its broad absorption capacity in the UV–Vis range, together with its antioxidant and radical scavenging properties^6,16^.

However, published data on photoprotection resulting from epidermal melanin are contradictory, and the relationship between pigmentation and photoprotection may extend beyond epidermal melanin content^2,17^. Indeed, several previous studies have also shown photosensitizing properties of melanin after UVR exposure^2,8,10,18–20^. In contrast to eumelanin, l-cysteine-containing pheomelanin can become a photosensitizer after UV irradiation, leading to the formation of CPD even after UV exposure has ceased^2,6,10,21^. In 2015, Premi et al.^20^ referred to this type of CPD as dark CPD (dCPD). The proposed reaction mechanism termed “chemiexcitation” suggests that UV-induced reactive oxygen and nitrogen species lead to a peak of peroxinitrite (ONOO–) species in the cytoplasm and nucleus, which are capable of degrading and oxidizing melanin to melanin-carbonyls in a quantum triplet state with energy equivalent to UV photons^20,22,23^. The energy of excitation is transferred to the nearby DNA bases, resulting in dCPD formation. Although it remains understudied, dCPD has emerged as an important non-classical and novel pathogenic pathway in melanoma formation^17,22^.

In view of the recent findings on the importance of chemiexcitation and the controversial results on the dependence of skin color on DNA damage after UVR, the present study aimed at the evaluation of the relationship between UVR-induced skin damage and melanin photoprotection/photosensitization in tanned and light RHE, which serve as a model system for the in vivo situation. Melanin extraction and melanin content estimation by spectrophotometry were performed and validated first on ex vivo skin and translated to RHE models. DNA damage in the epidermis of the RHE was assessed immediately after irradiation and 24 h later using a broadband UV lamp and a far UV-C LED. The evaluation of the far UV-C source is justified by the fact that the use of irradiation < 240 nm as a disinfection tool has increased due to the corona pandemic, and is part of the objective of this work to cover radiation beyond solar irradiation. It is known that UV-C irradiation at < 240 nm penetrates only the upper epidermis and that the dependence of DNA damage on the melanin content is lower compared to UV irradiation, as demonstrated by Busch et al. in ex vivo human skin irradiation experiments^24^. In the present study, the formation of radicals immediately after irradiation was additionally evaluated, considering their contribution to the formation of dCPD. In addition, the melanin distribution was evaluated as a relevant factor for the protective properties of melanin. The melanin distribution in tanned RHE was assessed by two-photon excited fluorescence lifetime imaging (TPE-FLIM) based on its endogenous fluorescence^25,26^ and by Fontana-Masson staining. The previously evaluated distribution in RHE was compared with the in vivo melanin distribution for different skin types.

## Results

### Quantitative analysis of melanin in human epidermis and in RHE

**Figure 1:**
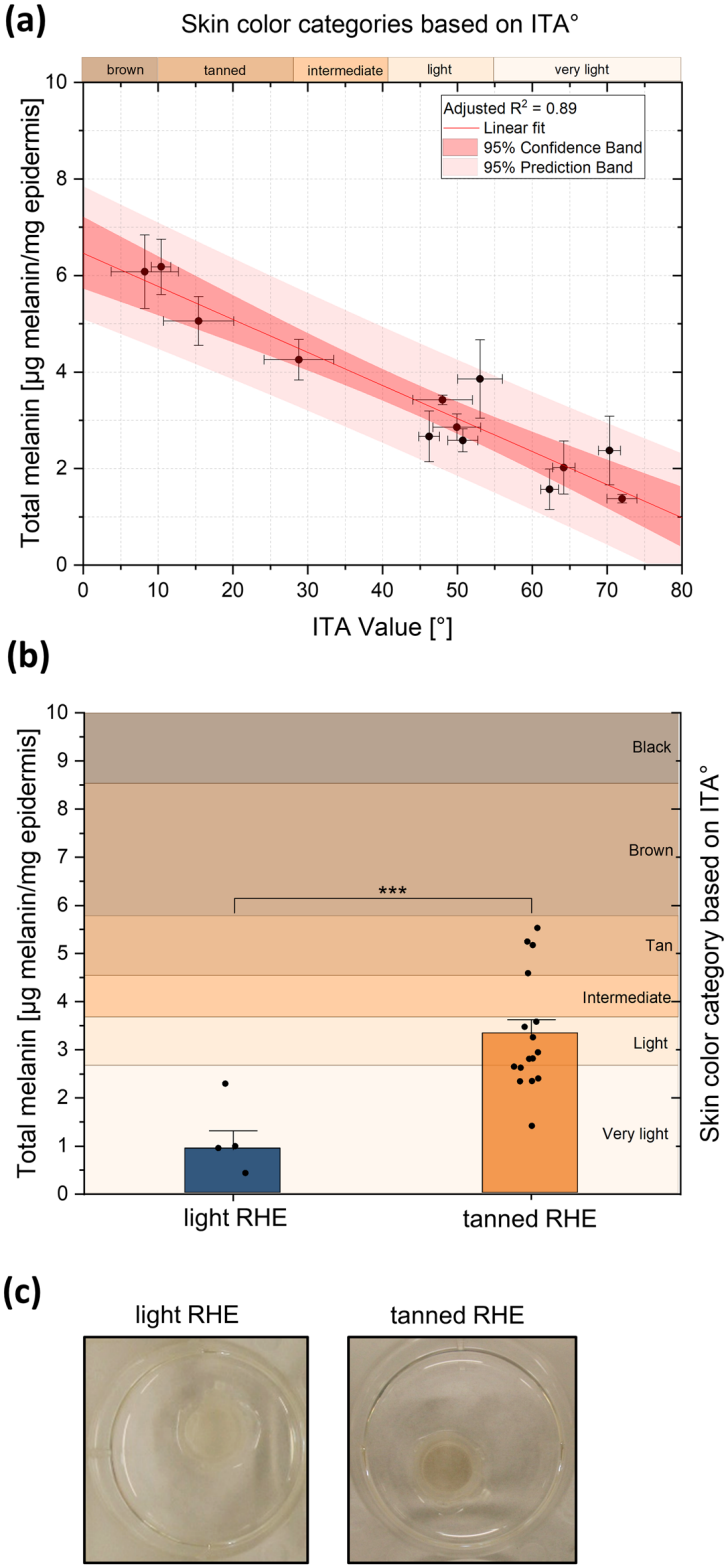
Melanin content of ex vivo skin samples and reconstructed human epidermis (RHE). (**a**) Correlation between skin melanin content [µg melanin/mg epidermis] (after an isolation procedure from ex vivo skin) and skin color measured as ITA [°] from ex vivo skin. The linear regression can be described by the functional equation y =  − 0.068x + 6.460 with R^2^ = 0.89. An increase in melanin content is reflected in a decrease in ITA° value. A statistically significant relationship exists between skin melanin content and ITA° as determined by ANOVA test (p < 0.05). Plot of linear regressions with respective 95% confidence band and prediction band. Data represents mean ± standard deviation. (**b**) Melanin content [µg epidermis/mg epidermis] after isolation procedure from RHE. An estimate of skin color according to the ITA° value classification system was calculated using the linear regression between melanin content and ITA° for ex vivo human skin. Tanned RHE achieved significantly higher melanin content compared to light RHE (Student’s t test), ***p < 0.001. For each ex vivo skin sample (n = 13 skin donors) n = 5 epidermis samples were analyzed (**a**), n = 5 for light RHE, and n = 16 tanned RHE (**b**). (**c**) Macroscopic view of tanned and light RHE after 15 days of culture at air–liquid interphase.

After the melanin extraction from excised human epidermis samples and RHE, the total melanin contents were estimated spectrometrically by measuring the absorbance of the sample at 500 nm. For ex vivo skin samples, the melanin content ranged from 1.38 ± 0.09 μg/mg (“very light” skin (Individual Typological Angle (ITA°) 72 ± 2°)) to 6.18 ± 0.57 μg/mg (“tanned” to “brown” skin (ITA° 10 ± 1°)). The validation of this method was performed by plotting the obtained melanin content versus the ITA° of ex vivo skin samples. Figure 1a shows that an increase in the melanin content results in a decrease in the ITA° values. According to the R^2^ and the statistical analysis of variance (ANOVA), the two variables showed a statistically significant correlation (R^2^ = 0.900; F(1;12) = 99.075; p < 0.05).

After validation of the optimal method to quantify melanin, the melanin content in RHE was evaluated (Fig. 1b). A total melanin content of 3.33 ± 0.30 μg melanin/mg epidermis was determined for the tanned RHE and 0.92 ± 0.40 μg melanin/mg epidermis for the light RHE. Student's t-test for paired samples showed significant differences (p < 0.001). With the melanin content of each RHE model, an estimation of the ITA° as well as the color category (according to the ITA° classification system^27,28^) were calculated for the RHE, using the linear regression between melanin content and ITA° from ex vivo human skin (Fig. 1a). Although the initial sources were different, a similar composition was assumed in both types of epidermis. The light RHE from cells of Caucasian donors (epiCS®-M /AC) corresponded to "very light" skin (approximated ITA° = 84 ± 6°) and the tanned ones from cells of Afro-American donors (epiCS®-M /AA) to “light” skin (approximated ITA° = 46 ± 4°) (Fig. 1c).

### Immunohistochemical assessment of DNA damage

**Figure 2:**
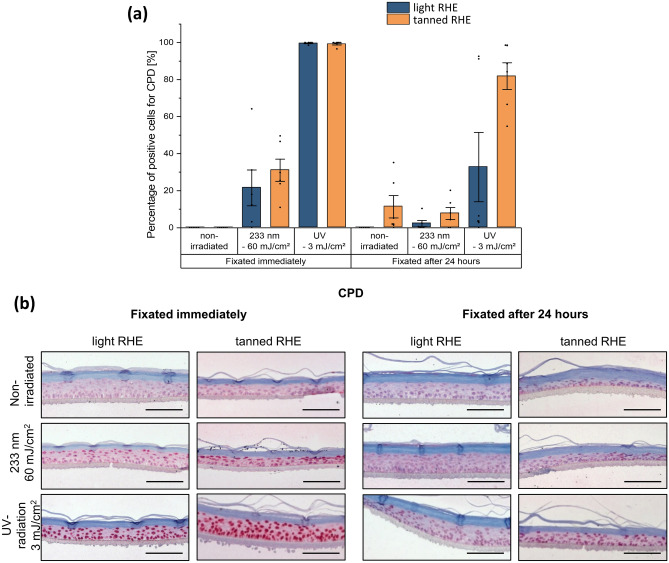
UV-induced cyclobutane pyrimidine dimers (CPD) in reconstructed human epidermis (RHE). (**a**) DNA damage, as percentage of cells with CPD formation, of light RHE (blue) and tanned RHE (orange), determined by immunofluorescence, fixated directly and 24 h after UV irradiation (233 nm–60 mJ/cm^2^ and UV—3 mJ/cm^2^). The non-irradiated sample serves as negative control and shows no damage when the samples were immediately fixated. Irradiation with 3 mJ/cm^2^ of UV induced the most DNA damage, significantly higher than damage after irradiation with 60 mJ/cm^2^ of 233 nm. Assessed immediately and 24 h post-irradiation, there is a reduction in CPD formation. Tanned RHE showed higher, although not significant, DNA damage compared to light RHE when fixated immediately after 233 nm irradiation and when fixating 24 h independent of the applied irradiation. Data of n = 6 biopsies (n = 3 models for each type of RHE). Data represents mean ± SEM. (**b**) Representative images of immunohistochemical detection of CPD positive cells in light and tanned RHE. Positive cells are stained in dark red. Samples irradiated with UV—3 mJ/cm^2^ resulted in stronger damage throughout the whole epidermis than those irradiated with 233 nm–60 mJ/cm^2^, where damage was concentrated on the uppermost layer of the epidermis. Scale bar: 100 μm.

DNA damage was evaluated by immunohistochemical staining of samples fixated immediately and 24 h after different types of UVR. For quantitative assessment, the data were expressed as the percentage of positive cells with CPD formation (% CPD) in relation to the total number of cells in the microscopic images (Fig. 2a). As the percentage of 6-4PP+ was close to 0% in all groups analyzed, the measure of DNA damage was based only on the percentage of CPD.

The DNA damage assessment immediately after irradiation showed that UV irradiation at 3 mJ/cm^2^ induced almost 100% CPD positive cells, regardless of the type of pigmentation of the RHE, and the percentage was significantly higher compared to the other two groups (Table 1, p = 0.048 for UV *vs.* untreated group or UV vs. 233 nm for both types of models, Fig. 2b). Samples exposed to UV–3 mJ/cm^2^ resulted in a damage throughout the whole epidermis, while samples exposed to 233 nm–60 mJ/cm^2^ resulted in a damage concentrated on the uppermost layer of the epidermis (Fig. 2b). After 233 nm irradiation, a significant difference in skin damage compared to untreated models was observed in tanned models (p = 0.048) but not in light models (p = 0.360). The non-irradiated models showed no presence of CPD cells, with the exception of the tanned models fixated 24 h later. After far UV-C irradiation at 233 nm with 60 mJ/cm^2^, the immediate damage was lower in light RHE than in tanned RHE.

**Table 1:** Multiple pairwise comparisons with manual Bonferroni correction after a Wilcoxon–Mann–Whitney test of % CPD between groups to assess the effect of UV irradiation wavelength on DNA damage (immediately and 24 h after exposure for each type of RHE), DNA damage repair 24 h after exposure and the influence of melanin, *p < 0.05. NI = non-irradiated, UV = irradiated with UV radiation, 233 nm = irradiated with far UV-C, 24 h = fixation 24 h after irradiation.

Effect	RHE type	Pairwise comparison	Significance	Correction
Immediately after irradiation	Light RHE	NI *vs.* UV	0.002	0.048*
		233 nm *vs.* UV	0.002	0.048*
	Tanned RHE	NI *vs.* 233 nm	0.002	0.048*
		NI *vs.* UV	0.002	0.048*
		233 nm *vs.* UV	0.002	0.048*
24 h after irradiation	Tanned RHE	NI *vs.* UV	0.002	0.048*
		233 nm *vs.* UV	0.002	0.048*
Repair	Light RHE	UV *vs.* UV 24 h	0.002	0.048*

To further investigate possible repair mechanisms (enzymatic or apoptosis) after irradiation, models were incubated at 37 °C for 24 h in culture medium after irradiation*.* A reduction in skin damage after 24 h compared to DNA damage directly after irradiation was observed in all treated groups (Fig. 2), although the reduction was significant only in the light models irradiated with UV (p = 0.048). When evaluating the effect of the presence of melanin in the RHE, tanned models showed greater DNA damage than light models in all groups evaluated 24 h after irradiation. The high variability between the RHEs made it difficult to obtain significant differences between tanned and light models in each type of irradiation, even though there is a clear trend in the individual results (Table 1).

### Radical formation

**Figure 3:**
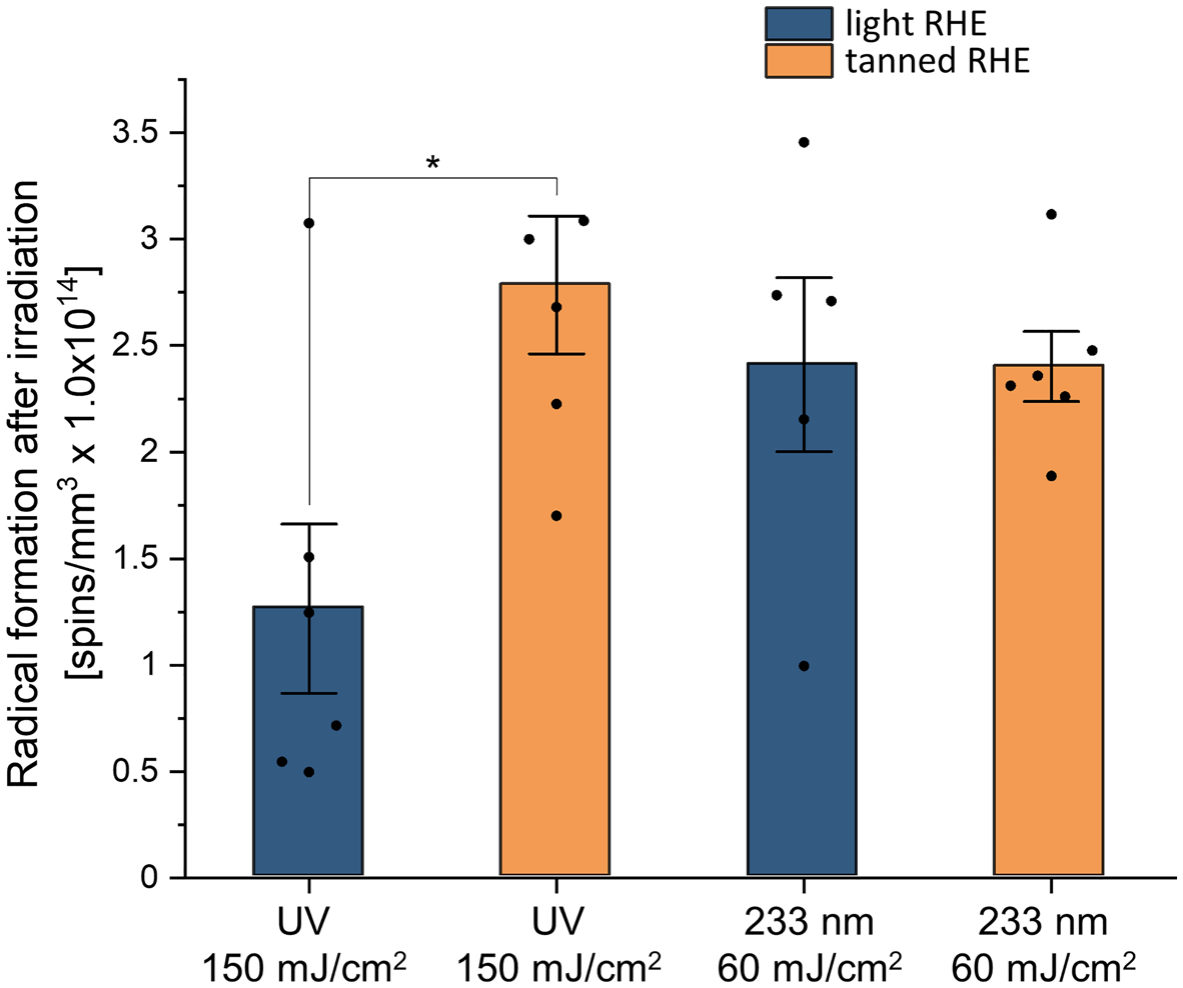
UV-induced free radical formation in reconstructed human epidermis (RHE). Radical formation directly after irradiation with 150 mJ/cm^2^ of UV radiation and 60 mJ/cm^2^ of far UV-C at 233 nm in light and tanned RHE. Data show the spin concentration (spins/mm^3^ × 10^14^) measured by electron paramagnetic resonance (EPR) spectroscopy. Significantly higher radical formation is induced in tanned RHE after UV irradiation compared to light RHE, while the formation after far UV-C exposure was similar for both types of RHEs (one way ANOVA with Bonferroni correction for post hoc test). n = 6 biopsies (n = 3 for each type of RHE). Data represents mean ± SEM, *p < 0.05.

Immediately after UV irradiation with 150 mJ/cm^2^ and far UV-C irradiation with 60 mJ/cm^2^, free radical formation could be measured in both types of RHE (Fig. 3). The tanned models showed directly after UV irradiation a significantly higher production (p = 0.022) of free radicals after UV irradiation, compared to the light models. Directly after far UV-C irradiation, this effect was not observed.

### Melanin distribution

**Figure 4:**
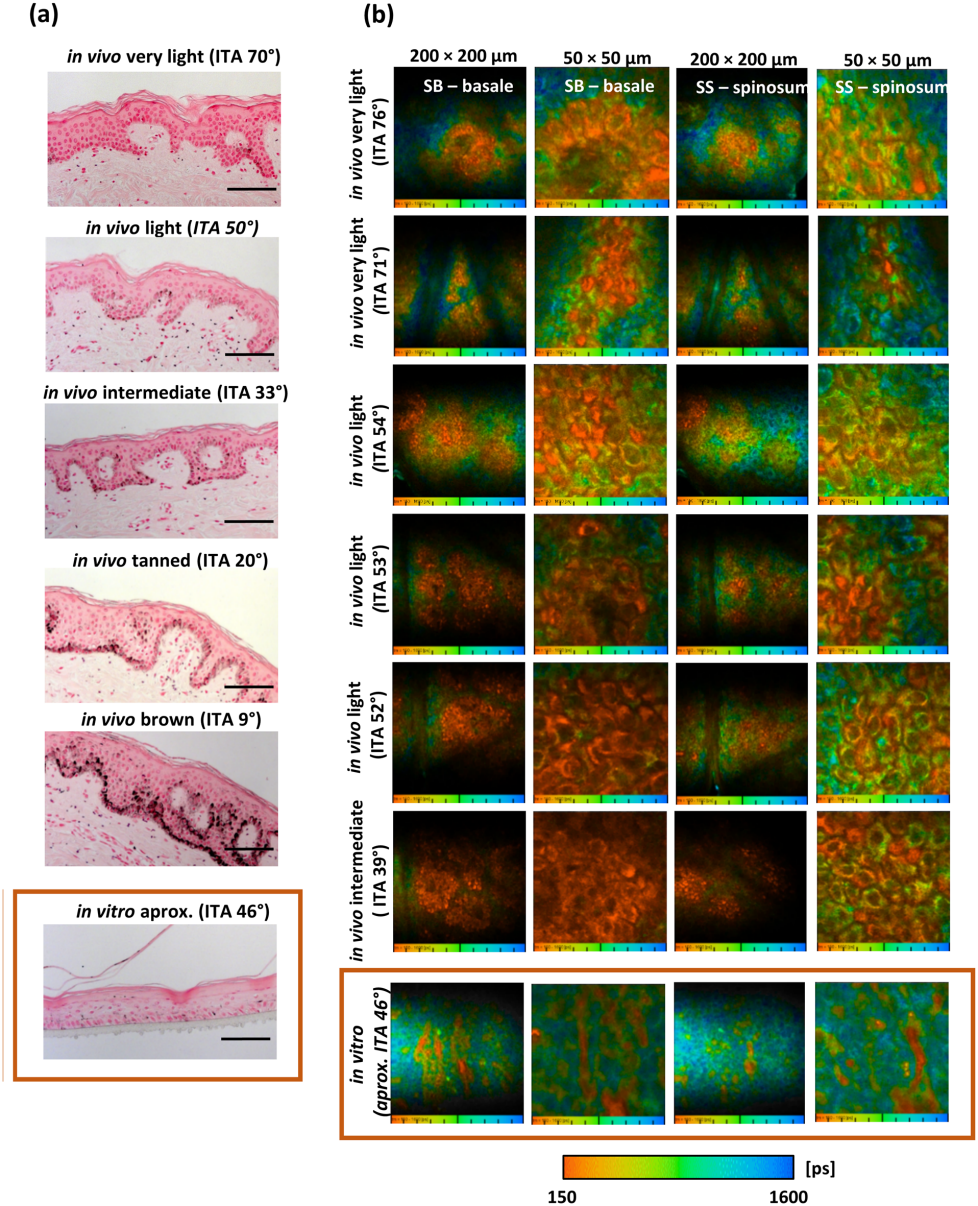
Epidermal melanin distribution by Fontana–Masson staining and two-photon excited fluorescence lifetime imaging (TPE-FLIM). (**a**) Representative images of Fontana–Masson staining of human skin (in vivo) and tanned RHE (in vitro). An example of each skin type is shown with the corresponding Individual Typology Angle (ITA°): “very light” skin, “light” skin, “intermediate” skin, “tanned” skin and “brown” skin. Melanin content increases with lower ITA°. Independent of the skin type, melanin in vivo is located perinuclear within basal cells, whereas melanin in tanned RHE remains extracellular. Scale bar: 100 μm. (**b**) TPE-FLIM (transversely) of in vivo human skin and tanned RHE. Melanin distribution in the stratum basale (SB) and the stratum spinosum (SS) is visualized continuously in false colors for lifetimes between 150 (orange) and 1600 ps (blue). Melanin-containing cells (stained orange) were masked by the characteristic short fluorescence mean lifetime of melanin (Tm < 480 ps). Both general (200 × 200 µm) and magnified (50 × 50 µm) images were acquired. The distribution of melanin in different skin types is shown with the corresponding individual typological angle (ITA°) and contrasted with the distribution of melanin in tanned RHE. In in vivo human skin, melanin-containing cells are concentrated in the basal cells and increase with decreasing ITA°. In volunteers with lighter skin, an increase in non-melanized (stained blue) or moderately melanized cells was evident, although a homogeneous distribution was maintained, shown in the images as a smooth transition between lower mean lifetime τ_m_ and higher mean lifetime τ_m_. In contrast, in tanned RHEs, melanin is concentrated in a limited number of extracellular sites, while most cells do not contain melanin. n = 6 spots were acquired from ventral forearms of volunteers with “very light” (n = 2), “light” (n = 3) and “intermediate” skin (n = 1) and from tanned RHE (n = 3).

Fontana-Masson staining allows to visualize the melanin distribution within the entire epidermal layer. The higher the skin pigmentation, the higher the pigment density in the basal layer of the epidermis. Figure 4a illustrates how melanin was perinuclearly localized, covering the surface of keratinocytes in all skin types. This pattern was not identified in tanned RHE (Fig. 4a, framed image in the orange box). Although melanin was detected in the epidermal layer, it was not localized within the epidermal keratinocytes.

Due to the fluorescence properties of melanin, its distribution in the epidermis can be further visualized by TPE-FLIM. Two-photon excited fluorescence intensity decays of in vivo human epidermis of volunteers with “very light” (n = 2), “light” (n = 3), and “intermediate” skin (n = 1) were analyzed by FLIM methods and compared with fluorescence intensity decays of tanned RHE (*n* = 3) (Fig. 4b). Melanin-containing cells in the stratum basale and spinosum were masked using the short fluorescence lifetime characteristic of melanin (mean fluorescence lifetime—*τ*_*m*_ < 480 ps, as well as short fluorescence lifetime component—*τ*_*1*_ < 150 ps and the respective amplitude a_1_ [%] > 92%, see Eq. (1)

1 $${\tau }_{m}= \frac{{a}_{1}{\tau }_{1}+ {a}_{2}{\tau }_{2 }}{{a}_{1}+ {a}_{2}},$$

and are shown as orange cells in Fig. 4b.

#### Melanin distribution within the basal cells

As expected, FLIM analysis in vivo with the mask *τ*_*m*_ < 480 ps allowed to identify the presence of melanin-containing and non-melanin-containing basal cells (Fig. 4b). Thus, differences in melanin area (shown as orange cells on the color scale) were detected between subjects with different ITA°, with an increase in melanin area at lower ITA°. In individuals with lighter skin, an increase in non-melanized (shown as blue cells on the color scale) or moderately melanized cells was evident, although a homogeneous distribution is maintained, shown in the images as a smooth transition between lower mean lifetime *τ*_*m*_ and higher mean lifetime *τ*_*m*_. In contrast, in tanned RHE (Fig. 4b, framed images in the orange box), the transition between melanin-containing and non-melanin-containing cells is lost, and in most cases, melanin is only concentrated in certain spots of the basal layer, while most cells show absence of melanin.

**Figure 5:**
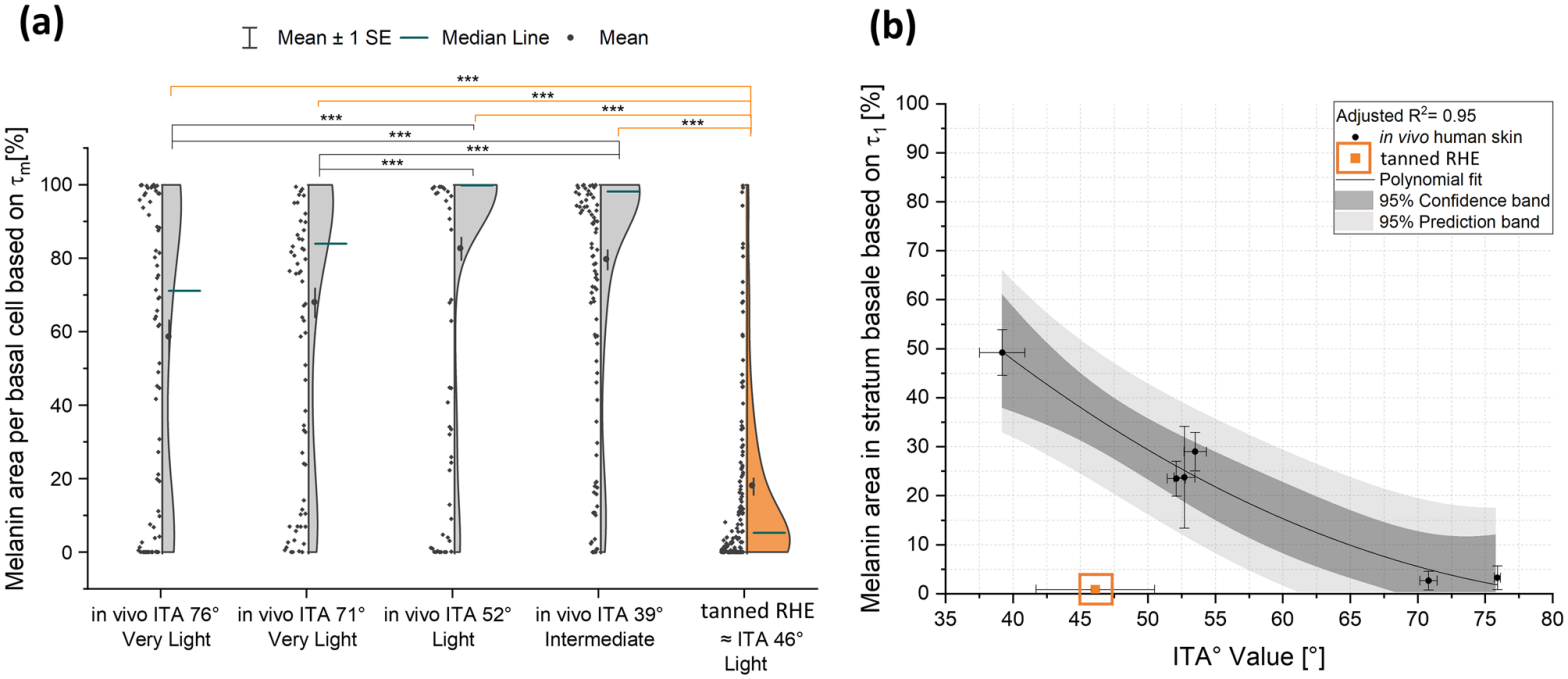
Intracellular and stratum basale melanin distribution using multiphoton FLIM analysis. (**a**) Modulation of intracellular melanin distribution in basal cells with skin color ITA° classification system for in vivo human skin and tanned RHE. Intracellular melanin distribution, the cell area (excluding nuclei) covered by melanin in each defined basal cell (extracted from magnified FLIM images at 50 × 50 µm, Fig. 4) was evaluated for volunteers with “very light” (n = 2), “light” (n = 1) and “intermediate” skin (n = 1) and for tanned RHE (n = 3). Melanin mask of τ_m_ < 480 ps. Regardless of skin type, melanin in vivo is homogeneously distributed: while darker skin types contain a high melanin area in most cells, lighter skin expresses a lower melanin area, but melanin is still present in most basal cells. In contrast, in tanned RHEs, the pigment is concentrated in a limited number of extracellular sites, while most cells do not exhibit melanin. Significant differences were found between the distribution of melanin in RHE and in all the human skin samples analyzed (Kruskal–Wallis-Test with Bonferroni correction), ***p < 0.001. (**b**) Correlation between melanin area in stratum basale [%] and skin color measured as ITA° [°] from measurements on in vivo human skin and tanned RHE. Melanin area in stratum basale is defined as the ratio between melanin pixels to stratum basale pixels (extracted from FLIM images at 200 × 200 µm, Fig. 4b). Melanin mask of τ_1_ < 150 ps and a1 [%] > 92%. The melanin area was evaluated for volunteers with “very light” (n = 2), “light” (n = 3) and “intermediate” skin (n = 1) and from tanned RHE (n = 3). A statistically significant relationship exists between melanin area and ITA° value as determined by ANOVA test (p < 0.05) and can be described by a second order polynomial fit with intercept = 164.09, B1 = -3.76 and B2 = 0.02, and R^2^ = 0.95. Plot of polynomial regression with respective 95% confidence band and prediction band. Data represents mean ± standard error of the mean.

To address the question of intracellular melanin distribution (melanin mask of τ_m_ < 480 ps), the cell area (excluding nuclei) covered by melanin in each defined basal cell (magnified images at 50 × 50 µm) was evaluated for volunteers with “very light” (n = 2), “light” (n = 1) and “intermediate” skin (n = 1) and for tanned RHE (n = 3) and is shown in Fig. 5a. Under in vivo conditions, multiphoton FLIM could highlight the homogeneous distribution of melanin in the basal cells of all skin types. In volunteers with darker skin type (“intermediate” skin), the median of the area covered by melanin was 100%, indicating that most cells are covered by a large area of melanin. For the subject with an ITA 52° (“light” skin), the average melanin area was 82.7 ± 3.0%, and for the subject with an ITA 39° (intermediate), it was 97.5 ± 0.8%. In individuals with lighter skin type, the area covered by melanin in the cells was not concentrated in a specific area, but the relative area size was distributed over a wider range, between 0 and 100%, as previously demonstrated by Vicente et al.^11^. The mean melanin area per cell for the subject with an ITA 76° (“very light” skin) was 58.1 ± 4.4%, ITA 70° (“very light” skin) was 68.1 ± 3.9%. As a result, there are approximately equal proportions of fully melanized keratinocytes, moderately melanized keratinocytes, and keratinocytes without melanin. Hence, even in individuals with a lighter skin (“very light” skin) with low melanin content, the pigment is transported and distributed homogeneously in most keratinocytes. Compared to the distribution of the melanin area in in vivo skin, the melanin area in tanned RHE cells did not resemble any of the in vivo distributions evaluated (Fig. 5a). Homogeneity test for variances and mean comparison showed significant differences between the tanned RHE and all in vivo measurements (p < 0.001), even if the approximated ITA of 46° for the RHE models would correspond to a “light” skin, similar to the subject with an ITA 52° (“light” skin) or with an ITA 39° (“intermediate” skin). The melanin area in most cells of tanned RHE is close to 0% (median 8.1%), and the proportion of cells with higher percentages of melanin area is limited. The mean melanin area for tanned RHE is 18.2 ± 2.0%.

#### Melanin distribution in the stratum basale

The melanin distribution was further analyzed in the stratum basale (200 × 200 µm overview images) and correlated with the skin constitutive pigmentation measured by ITA° for validation. Figure 4b shows examples of the TPE-FLIM color maps from the stratum spinosum and stratum basale of six volunteers with different ITA° as well as of the tanned RHE. An increase in melanin area is clearly identified in the deeper layers, attributed to the accumulation of this pigment in the stratum basale. However, when correlating the melanin area obtained by the mask *τ*_*m*_ < 480 ps with the ITA°, there was no model that significantly fits for this dependence. Due to the presence of other skin fluorophores that interact with melanin and contribute to the long-lived *τ*_*2*_ fluorescence, the correlation between skin pigmentation and fluorescence intensity defined by τm is affected^25,29^, thus, disabling the use of this mask in this case. The best way to correlate skin constitutive pigmentation with melanin fluorescence was to associate it with *τ*_*1*_*,* as previously demonstrated by Dancik et al.^29^. For the current study, τ_1_ was fixed at < 150 ps and the amplitude was varied to obtain the best correlation with constitutive pigmentation. The melanin mask with τ_1_ < 150 ps and a_1_ [%] > 92% is the one that best characterizes the correlation between melanin distribution and skin pigmentation by a second order polynomial fit with intercept = 164.09, B1 =  − 3.76 and B2 = 0.02. According to the R^2^ and the statistical ANOVA analysis, the two variables showed a statistically significant correlation (R^2^ = 0.973; F(1;5) = 53.498; p < 0.05).

This pattern was not observed in the tanned RHE data. Figure 5 once again showed a completely different distribution for the models. Although melanin extraction showed that tanned RHE had a higher melanin content than most of in vivo skins analyzed, the melanin area detected in the basal layer was close to zero (0.81 ± 0.22%) and fell outside the regression model.

## Discussion

Melanin is involved in many physiological processes in the human organism. In addition to its function as a skin pigmentation agent, melanin has been attributed functions such as protection against solar radiation, antioxidant properties, but also photosensitizing properties and a direct relationship to pathological skin processes such as melanoma^8,10,30^. Previous studies have shown that melanin and its chemiexcitation process are associated with melanoma initiation, progression, metastasis, and resistance to targeted therapies^8,22^. Melanin is therefore considered a double-sided molecule whose effects on the skin are still not fully understood^10,30,31^. To improve knowledge of the factors that influence the properties of melanin in the skin, the relationship between skin pigmentation and the incidence of UV-induced DNA damage needs to be further explored. Along with melanin content and distribution, UV-induced CPD immediately and 24 h after exposure and UV-induced ROS immediately after exposure were evaluated in tanned and light three-dimensional reconstructed human epidermal models. To compare the melanin distribution under in vivo conditions with the previously evaluated distribution in epidermal models, healthy volunteers of different skin types were assessed.

First, the method for extraction and quantification of melanin from ex vivo skin samples was established and was shown to be transferable to tanned and light RHE. Based on the melanin content, light RHEs showed an approximate ITA of 84° (which corresponds to “very light" skin), while tanned RHEs showed an ITA of 46° (which corresponds to “light” skin). Based on the skin color ITA° classification system, it is noticeable that the final pigmentation achieved by tanned RHEs was not high enough to represent darker skin types. Previous studies have already identified the technical challenges of obtaining cell stocks of primary epidermal keratinocytes and melanocytes especially from dark skin donors^32^. Cell populations proliferate very slowly and the communication between melanocytes and keratinocytes is difficult to simulate, thus hindering sufficient melanin production, a proper pigment transfer mechanism and correct positioning of melanin in the basal layer^13,32^. However, in the current study, the total melanin content of tanned RHE was significantly higher than that of light RHE, by a factor of three.

UV-induced DNA damage results highlighted the sensitivity of the epidermal models to UVR and their ability to repair the induced DNA damage (Fig. 2). Both RHEs produced a significant increase in immediate DNA damage at both wavelengths with a reduction 24 h after UV-exposure. According to the results targeted for % CPD, UV radiation induced the most DNA damage even at low doses (1/10 minimal erythema dose—MED, 3 mJ/cm^2^), with significant differences compared to the untreated RHE and far UV-C with 60 mJ/cm^2^ irradiated RHE. The results are in agreement with those reported by Zwicker et al*.* in which irradiation studies were carried out on human epidermal models lacking melanocytes, showing 94% positive keratinocytes after 3 mJ/cm^2^ of UV irradiation and only negligible CPD damage after irradiation with 60 mJ/cm^2^ at 233 nm^33^. Irradiation experiments on ex vivo human skin showed nearly 70% CPD-positive cells after 10% MED UV and 30% CPD-positive cells after 60 mJ/cm^2^ far UV-C^24^. Immunohistochemical staining also revealed a consistent penetration depth of UV radiation in the tanned and light RHE. As previous studies showed, UV-B and UV-A radiation are able to penetrate the entire epidermis and reach basal stem cells; this energy is readily absorbed by nucleic bases, resulting in DNA lesions immediately after irradiation^34^. Far UV-C radiation, which is strongly absorbed by proteins of the stratum corneum, only penetrates the superficial layers of the epidermis, reducing DNA damage in the basal cells^34^.

Although higher skin pigmentation is associated with increased skin protection against solar radiation, and melanin analysis showed quantitative differences between tanned and light RHE, all data in the current study revealed that less DNA damage is induced by lower epidermal pigmentation (Figs. 2, 3). Studying DNA lesions from both UV irradiation types after direct fixation showed similar, if not more, CPD lesions in tanned RHE than in light RHE. This difference became even greater 24 h after exposure, where the tanned RHE tended to show more CPD formation in the UV and far UV-C groups examined. The possibility of increased damage to tanned models due to thickness variation was ruled out (data not shown), as the depth of both type of RHE was comparable. Hence, it could be hypothesized that the photoprotective factor associated with epidermal melanin is altered in RHE. Instead, it might be a possible UV-mediated photosensitizing effect of melanin that can result in DNA damage. As mentioned above, melanin has been implicated in both protective and UV-sensitizing properties. Under in vivo conditions in human skin, a protective role has been supported by several epidemiological and experimental evidence. However, some studies have yielded conflicting results, failing to simulate the inverse dependence of DNA damage on skin type. Recently, significant CPD formation has been described in mouse and in vitro models 2–3 h (retained up to 24 h) after exposure to broadband UV-A or narrowband UV-B via delayed melanin sensitization^2,35^. Therefore, the hypothesis that melanin may be a photosensitizer for the formation of dCPD in tanned RHE could not be excluded. The DNA lesions presumably arise as consequence of energy transfer from UV-induced excitation of melanin. Unlike eumelanin, pheomelanin is less photostable. When exposed to short wavelength UVR, it undergoes physicochemical modifications that decrease its protective effect and increase its pro-oxidative potential, creating a mutagenic environment^8^.

The formation of melanin induced-DNA lesions was further supported by measurements of UV-induced free radicals immediately after irradiation. Tanned RHE again showed quantitatively higher free radical production after exposure to 150 mJ/cm^2^ UV compared to light RHE. After exposure to 60 mJ/cm^2^ far UV-C, tanned and light RHE produced similar levels of free radicals. These wavelength-dependent variations may be explained by the penetration depth of radiation into the skin together with the melanin distribution in the basal layers^24,34^.

Mainly UV-A photons, but also UV-B photons from UV radiation, which are capable of penetrating the entire epidermis, come into contact with melanin. This contact leads to melanin sensitization, which ultimately triggers an increased production of free radicals in tanned RHE compared to light RHE. Far UV-C photons, on the other hand, reach only the upper layers of the epidermis where melanin is already dispersed, reducing the dependence of free radical formation on the melanin content^24^.

Thus, all results point to UV-induced, melanin-dependent mutational patterns in tanned RHE. It remains to be elucidated why melanin exerts a prooxidative effect in tanned RHE contrary to the well-known photoprotective effect under in vivo conditions. The effects described may be influenced by factors such as the type of melanin, its epidermal distribution and content^13^. The importance of the melanin distribution in the human epidermal keratinocytes has been recognized in the literature for many years, where supranuclear melanin caps absorb or scatter incident radiation energy to protect the nucleus from UV-induced DNA damage^36^. Indeed, Kobayashi et al. in 1998 showed a significant decrease in the formation of DNA lesions in basal cells with melanin caps compared with keratinocytes without such melanin distribution^15^. Fontana–Masson staining clearly shows the accumulation of melanin in the basal layer, covering the surface of the keratinocytes in all skin types included in the present study. Although the content of this pigment is minimal in “very light” skins, the perinuclear localization remains present. On the contrary, in tanned RHE, with a melanin content similar to that of skin with ITA 46°, the melanin detected tends to be localized extracellularly. However, due to low sensitivity and specificity of this staining^37^, the global and intracellular distribution of melanin was further evaluated using TPE-FLIM.

Multiphoton FLIM analysis has proven to be more sensitive in detecting global and intracellular melanin distribution^25,26^. First, the extracellular localization in the tanned RHE detected by Fontana-Masson staining was validated. Second, the tanned RHE displayed an inhomogeneous distribution in the basal layer of the epidermis, with accumulations of melanin at certain spots. As a result, although melanin was detected by spectrophotometry in the tanned models, melanin remained concentrated in certain cells and was not homogeneously distributed in neighboring cells. The differences found in the distribution of melanin in vivo and in RHE highlight its influence in determining the properties of melanin in the skin. Variations in the melanin distribution, and in particular the localized accumulation of melanin, may have led to increased melanin degradation after UV exposure, resulting in increased in situ generation of free radicals capable of causing DNA damage compared to in vivo conditions. It has been shown that after irradiation with UV-A or UV-B, melanin is oxidized, initiating a high formation of superoxide anions and hydrogen peroxide which, in turn, are capable of degrading melanin into fragments and, subsequently, decomposing it into triplet-excited products^38,39^. The importance of evaluating the degradation products as well as the type of radicals induced after exposure of melanin to UVR is recognized. Future research focusing on those aspects is needed.

The results presented in the current study once again demonstrated the photosensitizing properties of melanin in the skin after exposure to UVR, capable of generating higher levels of free radicals that ultimately lead to the induction of DNA lesions. An inhomogeneous and localized distribution in certain cells proved to be a determining factor. The biological implication of this pro-oxidative effect of melanin in the skin and in the pathogenesis of skin cancer in dark individuals deserves further investigation.

## Materials and methods

### Reconstructed human pigmented epidermal model (RHE)

Reconstructed human pigmented epidermal models were used to evaluate the effect of melanin after UV irradiation. The epiCS®-M epidermal models (Henkel AG & Co. KGaA, Düsseldorf, Germany) were cultured from normal human primary epidermal keratinocytes and melanocytes from Asian-Caucasian or Afro-American donors. RHE were then classified as tanned and light models, respectively. To assess melanin levels, a total of *n* = 5 samples of light RHE and *n* = 16 samples of tanned RHE were evaluated. The effects of UV irradiation were investigated by evaluating *n* = 36 RHE samples, including *n* = 18 samples of light RHE and *n* = 18 samples of tanned RHE.

Upon receipt, the RHE models were cultured for seven days in six-well plates at 37 °C, 5% CO_2_, and 95% humidity in epiCS ®-M culture medium. The medium was changed every two days. On the eighth day of culture, the melanin content was assessed, and the models were irradiated. The determination of pigmentation of in vitro models by using optical parameters is not feasible due to technical limitations when applying the electrode to the cell culture insert. A method for melanin extraction and quantification was established and validated on ex vivo human skin. Parameters such as ITA° of the RHE could also be estimated from these results.

### Ex vivo human skin

Melanin extraction and quantification on ex vivo human skin enables the estimation of the pigmentation type of the models, allowing for their classification based on melanin content. Ex vivo human skin samples were obtained from surgical residues of breast (*n* = 4) and abdominal (*n* = 9) reduction surgery from healthy subjects aged 24–63 years, of different ethnicities. All patients had given their informed written consent. All procedures and measurements were approved by the Ethics Committee of the Charité Universitätsmedizin Berlin (EA1/324/19) and complied with the Declaration of Helsinki. In the laboratory, subcutaneous tissue was removed down to the dermis using a scalpel (Aesculap AG, Tuttlingen, Germany), and the skin surface was cleaned using PBS solution (Gibco™, New York, USA). A Skin-Colorimeter CL 400 (Courage & Khazaka electronic GmbH, Cologne, Germany) was used to measure the skin pigmentation (ITA°). Ten consecutive measurements were performed on adjacent skin areas. Values were expressed in degrees [°] and were averaged. According to the ITA°, the skin types of the samples are classified into six skin groups, from very light to dark skin: “very light” > 55° > “light” > 41° > “intermediate” > 28° > “tan” > 10° > “brown” > 30° > “dark”^40^.

### In vivo human skin

Healthy volunteers were enrolled to evaluate the in vivo melanin distribution in different skin types using TPE-FLIM and Fontana–Masson staining. All studies were undertaken with the approval of the Ethics Committee of the Charité—Universitätsmedizin Berlin and the written consent from the volunteers in accordance with the Declaration of Helsinki and are registered in the German Clinical Trials Register (DRKS00028055). A Skin-Colorimeter CL 400 (Courage + Khazaka electronic GmbH, Cologne, Germany) was used to measure the skin pigmentation (ITA°). Three male and three female volunteers aged 26 to 34 years with “very light” (n = 2), “light” (n = 3) and “intermediate” skin (n = 1), were enrolled to evaluate the transversal melanin distribution by TPE-FLIM in the area of the inner forearm. Skin color measured by reflectance spectrophotometers allowed to validate of the FLIM parameters used to assess melanin distribution in the skin. In addition, to evaluate the longitudinal distribution of melanin by Fontana–Masson staining, a skin biopsy was taken from the lower back of four male and one female volunteers aged 21 to 38 years. One subject of each skin type (“very light”, “light”, “intermediate”, “tanned”, and “brown”). The biopsy was fixated in neutral buffered 4% formalin solution (Sigma # HT501128-4L, Merck KGaA, Darmstadt, Germany), embedded in paraffin, and stained with Fontana-Masson for histological detection of melanin.

### Melanin content

Ex vivo human skin was prepared and subjected to melanin extraction. The epidermis was first separated by a heat-separation procedure^41^. 8-mm (ø) punch biopsies (pfm medical, Kai Industries Co. Ltd., Oyana, Japan) of each skin sample were taken and placed on the hot plate at 60 °C for 1 min. The epidermis was then completely separated from the dermis using tweezers. Epidermal samples were dried for 1 h at room temperature and weighed into tubes. Samples (between 3 and 5 mg dry weight) were solubilized in SOLVABLE™ (PerkinEimer Inc., Waltham, USA) at a concentration of 10 mg/mL. The samples were homogenized using a TissueLyser II (QIAGEN, The Netherlands) for 10 min at 30 rs^−1^ with stainless steel beads and dissolved by subsequent heating in a boiling water bath for 1 h. For spectrophotometric characterization of the skin melanin, the absorption spectra of the resulting solutions were recorded in the range of 450 to 600 nm on a UV/VIS spectrophotometer Lambda 650 S (PerkinElmer LAS GmbH, Rodgau-Jügesheim, Germany). To estimate the total melanin (eumelanin and pheomelanin), the absorbance at 500 nm was analyzed^12,40,42^, and the melanin content was calculated by interpolation of the results with standard curves, generated by the absorbance of synthetic melanin standards dissolved in SOLVABLE™ (Sigma-Aldrich Chemie GmbH, Steinheim am Albuch, Germany). The results were normalized by the concentration of epidermal samples in SOLVABLE™ (10 mg/mL). For the RHE, the extraction and estimation of the melanin content required some adjustments. The insert membrane with the epidermis equivalent was cut from the cell culture insert and dried for 1 h at room temperature. Each model (approximately 2 mg dry weight) was solubilized in SOLVABLE™ at a concentration of 5 mg/mL. A cell culture insert membrane was used as control. Homogenization and spectrophotometric characterization were performed as previously described for ex vivo skin. Absolute concentrations were also obtained using synthetic melanin as a standard and normalized by the concentration of the epidermal samples (RHE) in SOLVABLE™ (5 mg/mL). For each ex vivo skin sample (*n* = 13 skin donors) *n* = 5 epidermis samples were analyzed. For light RHE *n* = 5 and for tanned RHE* n* = 16.

### UV irradiation

**Figure 6:**
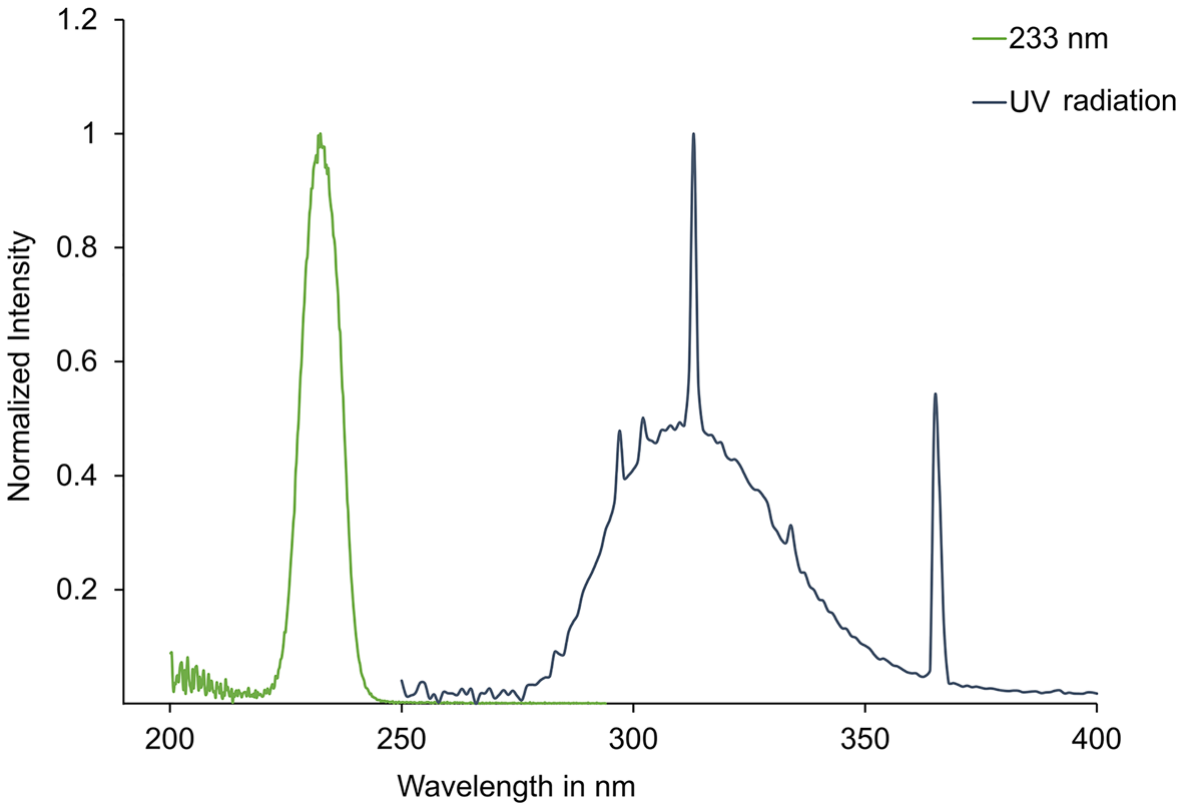
Emission spectra of applied UV light sources. The normalized spectra are shown for 233 nm (green), broadband UV-radiation (blue).

For the irradiation of the RHE, different types of UVR at different doses were investigated. Far UV-C radiation of 233 nm wavelength (0.041 mW/cm^2^, UV-C LED irradiation source with a short pass optical filter suppressing wavelengths > 240 nm, Ferdinand-Braun-Institute gGmbH, Berlin, Germany) at a dose of 60 mJ/cm^2^ was applied, which has been shown to be effective in reducing germs on the skin and wounds and is safe for the skin due to its low penetration into the deep layers of the epidermis. A broadband UV lamp containing the UV-B (280–315 nm; 50.69%) and UV-A (315–400 nm; 48.21%) fractions was used in this study (Fig. 6). The lamp used was model TH-1E from Cosmedico®, JW Sales GmbH, Stuttgart, Germany, with an intensity of 41 μW/cm^2^. The inclusion of UV-B and UV-A wavelengths allowed the assessment of direct DNA damage and free radical formation, respectively, due to their different effects on the skin^43^. To assess DNA damage, a dose corresponding to 1/10 MED (3 mJ/cm^2^ for skin type II^44^), a dose which is considered acceptable for the skin, was applied^34^. A dose of 150 mJ/cm^2^ of UV was used to assess UV-induced free radicals. The pursuit of a biological response to UV irradiation comparable to that of far UV-C irradiation in DNA damage and radical formation has led to variations in the doses used for UV irradiation. Non-irradiated RHE samples, served as negative control. Irradiance of radiation for 233 nm was measured with the UV radiometer SXL55 with a SiC UV-C sensor (sglux GmbH, Berlin, Germany), and an ILT 1400 radiometer photometer (International Light Technologies Inc., Peabody, MA, USA) for UV-B (SEL240) lamps. Data of *n* = 6 biopsies of *n* = 3 models for each type of RHE.

For irradiation, the RHE were placed in PBS previously heated to 37 °C to avoid possible photosensitivity of the culture medium. The negative controls were also transferred to 6-well plates with PBS to exclude any influence of this procedure and stored in parallel in the dark for the duration of the irradiation.

Normalized emission spectra of the used UV light sources are shown in Fig. 6.

### Analysis of DNA damage

To assess the effect of far UV-C and UV irradiation on skin cells, CPD and 6-4PP were evaluated in the epidermis. Two 4-mm (ø) biopsies were taken from each model, for a total of *n* = 6 biopsies per RHE type (*n* = 3 light RHE and *n* = 3 tanned RHE). *n* = 6 biopsies were then evaluated immediately and 24 h after irradiation, in each group: non-irradiated, 233 nm irradiation, and UV irradiation. The biopsies were taken from each model, fixated in neutral buffered 4% formalin solution (Sigma # HT501128-4L, Merck KGaA, Darmstadt, Germany) and embedded in paraffin (paraffin blocks) (Histosec™, Merck Millipore GmbH, Darmstadt, Germany). DNA damage was assessed immediately and 24 h after (reculture of RHE at 37 °C and 5% CO_2_) irradiation using the immunohistochemical approach described previously^45^. Tissue slides underwent immunostaining with specific antibodies, including anti-6-4PP (clone 64M-2, Cosmo Bio, USA) and anti-CPD (clone TDM-2, Cosmo Bio, USA). Detection of 6-4PP+ and CPD+ was achieved using Alkaline Phosphatase/RED, Rabbit/Mouse (Agilent Technologies, USA). Negative controls were implemented by omitting the primary antibody. Sections were examined using an AxioImager Z1 microscope (Carl Zeiss MicroImaging, Inc., USA) in a blinded manner. Manual counting of all positive epidermal cells was performed, and the results were expressed as the percentage of positive cells in a given image.

### Quantitative radical measurements

Quantitative analysis of UV-induced free radicals in RHE was performed by Electron paramagnetic resonance (EPR) spectroscopy on an X-band EPR spectrometer (Bruker Elexsys E500, BioSpin GmbH, Karlsruhe, Germany) using the spin marker PCA (3-(carboxy)-2,2,5,5-tetramethylpyrrolidin-1-oxyl) (Merck KgaA, Darmstadt Germany). Biopsies were obtained using 3-mm (ø) punch biopsies, treated with PCA, and exposed to the respective radiation sources: 60 mJ/cm^2^ 233 nm and 150 mJ/cm^2^ UV radiation (refer to “UV irradiation” section). The samples were then immediately analyzed by EPR, as previously described by Zwicker et al.^33^. Considering that UV-A radiation is primarily responsible for radical formation and that the UV lamp also includes a UV-B fraction^46^, the radiation dose had to be increased by about 5 MED (150 mJ/cm^2^) for skin type II to achieve a biological response comparable to that of 233 nm irradiation.

### Melanin distribution

Melanin distribution in human skin in vivo and in tanned RHE was assessed longitudinally and transversely by Fontana-Masson staining (see “In vivo human skin” section) and TPE-FLIM, respectively. Imaging by TPE-FLIM was performed using a two-photon microscope (DermaInspect™, JenLab GmbH, Jena, Germany) integrated with a tunable femtosecond Ti:sapphire laser (Mai Tai® XF, Newport Spectra-Physics GmbH, Darmstadt, Germany). The laser power was set to 45 mW. Images were acquired upon excitation at 760 nm and detection at 550 nm. In vitro and in vivo imaging was performed with an NA oil-immersion objective. A drop of saline solution was placed on the area of the skin or the RHE to be imaged. For the measurement, a metal accessory containing a cover glass with a drop of immersion oil was placed directly on the skin of the volunteers (volar side of the forearm) or the RHE. Images sized 200 × 200 µm and 50 × 50 µm were recorded on at least 6 spots per sample in the basal epidermal layer (above the papillary layer and collagen bundles) as well as in the stratum spinosum (10 µm from the stratum basale).

#### Data analysis

Fluorescence lifetime data analysis was performed using SPCImage software version 8.4 (Becker & Hickl GmbH, Berlin, Germany) assuming a bi-exponential decay and a binning value of 2. Melanin distribution was determined both within the cells of the stratum basale and the global distribution in the stratum basale. In the first case, 50 × 50 µm FLIM images (clearly delineated cells), were evaluated for the mean fluorescence lifetime (*τ*_*m*_), with melanin contributing significantly to very short fluorescence lifetimes (*τ*_*1*_). The use of the *τ*_*m*_, defined as the weighted average of the short lifetime components (*τ*_*1*_*)* and long lifetime component *(τ*_*2*_*)* in each image pixel and their respective amplitudes (*a*_*1*_*) *and (*a*_*2*_*)*, has been shown to correlate directly with the melanin concentration and was then justified for the evaluation of melanin distribution in the basal cells^24,26,29^.

2 $${\tau }_{m}= \frac{{a}_{1}{\tau }_{1}+ {a}_{2}{\tau }_{2 }}{{a}_{1}+ {a}_{2}}.$$

The images were exported in tif format and further processed for melanin distribution analysis using Fiji/ImageJ 1.53q (W. Rasband, NIH, USA). A region of interest of 400 × 400 pixels was defined and the manual segmentation of each of the defined cells was performed. The melanin area in each cell was expressed as the ratio of the number of melanin pixels to the number of epidermal cell pixels (excluding the nucleus) and was calculated using Matlab software (MathWorks, Inc., Natick, MA, USA). Pixels with *τ*_*m*_ < 480 ps would correspond to melanin pixels, as described before by Pena et al. in a recent publication^26^.

For the second case, the overview of the melanin distribution in the stratum basale, 200 × 200 µm FLIM images were processed by applying different thresholds to create the ideal melanin mask based on *τ*_*m*_ as well as *τ*_*1*_ and *a*_*1*_. For *τ*_*m*_, pixels with *τ*_*m*_ < 480 ps would correspond to melanin pixels. For *τ*_*1*_, a mask of *τ*_*1*_ < 150 ps was set, and the amplitude *a*_*1*_ was varied between 80 and 100 until the best correlation model with skin pigmentation was obtained. The melanin area in the stratum basale is defined as the ratio of the number of melanin pixels to the number of total pixels and is calculated using Matlab software.

### Statistical analysis

Statistical analyses were performed using IBM SPSS® Statistics 26 software (IBM, Armonk, N.Y., USA). For multiple measurements, a mean with corresponding standard error of the mean was calculated (MW ± SEM) and a test for outliers was performed. The relationship between spectrophotometric melanin-related descriptor (melanin content) and skin color optical parameter by ITA° was described graphically using a scatter plot and a linear regression model. The relationship between melanin area in the stratum basale (measured by TPE-FLIM) and ITA° was described graphically using a scatter plot and a polynomial regression model. The 95% confidence and prediction intervals were presented. The strength of the relationship was characterized by the coefficient of determination R^2^ (the square of the correlation coefficient) and tested for significance by ANOVA.

The Shapiro–Wilk test was used to test data for normal distribution and the Levene test for equality of variances. Two-tailed Student’s *t*-tests were performed for mean comparisons of the melanin content. For radical formation results, multiple comparisons of means were performed using a Kruskal–Wallis ANOVA, followed by Bonferroni post hoc tests. Pairwise comparisons for more than three groups (DNA damage) with non-parametric results and a significant difference under Kruskal–Wallis ANOVA were performed using multiple Wilcoxon-Mann–Whitney tests with manual Bonferroni correction. A p-value < 0.05 was chosen to indicate a significant difference. Data visualization as well as determination of regressions was performed using OriginPro® version 2019b (OriginLab Corporation, Northampton, Massachusetts, USA).

## Data Availability

Data that support the findings of this study are available from the corresponding author Martina C. Meinke (martina.meinke@charite.de) upon reasonable request.
